# Supplementary material for: Online activities as risk factors for Problematic internet use among students in Bahir Dar University, North West Ethiopia: A hierarchical regression model
Source: PLoS One. 2020 Sep 11;15(9):e0238804. doi: 10.1371/journal.pone.0238804 (PMC7485847; doi:10.1371/journal.pone.0238804)
Supplement: S1 File — (DOCX) [file pone.0238804.s001.docx]

# Additional file 1

## Survey Questionnaire

The purpose of this questionnaire is to collect data on students’ internet use behavior and purposes of internet use in Bahir Dar University. The data will help to know the levels of internet, major purposes of internet use, and identify antecedents of internet use among university students. The findings will provide relevant information for university authorities and other concerned bodies on how well students are using the internet and introduce regulatory mechanisms to limit the usage of potentially addictive online internet applications and promoting responsible use of the internet. The information is needed for research purpose. The information given will be kept confidential.

Thank you in advance for your participation

Respondent ID _________ Faculty ________________________

**Part I. Background characteristics of respondents**

| Please complete the following questions about you and your family | | |
| --- | --- | --- |
| S/N | Variable descriptions | Answers |
| 1 | Please encircle your sex | 1. Female 2. Male |
| 2 | Write your age in completed years | Age _________ |
| 3 | Religion | 1. Orthodox 2. Muslim 3. Protestant 4. Catholic 5. Others, specify |
| 4 | College/faculty you joined in the university | 1. Social science 2. Engineering 3. Law and land administration 4. Agriculture 5. Textile |
| 5 | Year spent in the university | 1. First year 2. Second year 3. Third year 4. Fourth year and above |
| 6 | Your grade point average in the university | CGPA ___________ |
| 9 | Estimated average annual income of your parents? | ETB : ________________ |
| 10 | Perceived living condition of your parents: | 1. Poor 2. Medium 3. Better off |
| 11 | Your parent’s residence? | 1. Urban 2. Rural |
| 12 | When you start to use the internet? | 1. At primary school (Grade 1-8) 2. At secondary (9 – 12) 3. After joining university |

**Part II. Internet use behavior questions**

| For the following internet use questions, mark “X” on the alternative that you think better explains you. | | | | | | |
| --- | --- | --- | --- | --- | --- | --- |
| S/N | Questions to assess the level of internet use | Alternative responses  1= Never  2= Rarely  3= Occasionally  4= Often  5= Always | | | | |
|  |  | 1 | 2 | 3 | 4 | 5 |
| 1 | How often do you find yourself stay online longer than you initially intended to be online? |  |  |  |  |  |
| 2 | How often do you log in and use internet at class while your instructor is giving his/her lecture? |  |  |  |  |  |
| 3 | How often do you prefer the excitement of being online and chatting with online friends over the intimacy and enjoyment with your friends and families in person? |  |  |  |  |  |
| 4 | How often do you form/establish new online relationship with online users? |  |  |  |  |  |
| 5 | How often do your significant others in your life complain about the amount of time you spend online? |  |  |  |  |  |
| 6 | How often do your school work and academic achievements suffer because of the amount of time you spend online? |  |  |  |  |  |
| 7 | How often do you log in to and use internet immediately after you wake up from your bed in the morning time before doing anything else? |  |  |  |  |  |
| 8 | How often do you miss class to chat with your online friends and to maximize your time on online activities? |  |  |  |  |  |
| 9 | How often do you become defensive or secretive when anyone asks you what you do online? |  |  |  |  |  |
| 10 | How often do you use soothing thoughts of internet as a way of escaping from problematic experiences in life or relieving from unhappy moods like helplessness or depression? |  |  |  |  |  |
| 11 | How often do you find yourself anticipating the time you will go online again? |  |  |  |  |  |
| 12 | How often do you fear that life without the internet would be boring, empty and joyless? |  |  |  |  |  |
| 13 | How often do you feel unhappy, moody or act annoyed when someone bothers you while you are online? |  |  |  |  |  |
| 14 | How often do you lose sleep due to being online late at night? |  |  |  |  |  |
| 15 | How often do you feel preoccupied with using internet or think about previous online activity when you are offline? |  |  |  |  |  |
| 16 | How often do you find yourself “saying just a few more minutes” to be offline when you are online? |  |  |  |  |  |
| 17 | How often do you try to cut down the amount of time you spend online and failed to be successful in doing so? |  |  |  |  |  |
| 18 | How often do you try to lie your family members, friends or any other significant others of you to hide your extended time involvement with the internet? |  |  |  |  |  |
| 19 | How often do you feel unhappy, worried or moody when your online friends are offline while you are online to chat with them? |  |  |  |  |  |
| 20 | How often do you feel depressed, moody or nervous when you are offline, which goes away when you back online? |  |  |  |  |  |

**Part III. Contributing factors for Internet use behavior**

**Parental support**

| We are interested in how you feel about the following statements. Read each statement carefully. Indicate how you feel about each statement and mark “X” on the alternatives you think better explains you. | | | | | |
| --- | --- | --- | --- | --- | --- |
| S/N | Questions to assess the level of parental support | Alternative responses  1= Strongly disagree  2= Disagree  3= Agree  4= Strongly agree | | | |
|  |  | 1 | 2 | 3 | 4 |
| 1 | I count on my parents when things go wrong |  |  |  |  |
| 2 | I get the emotional help and support I need from my parents |  |  |  |  |
| 3 | My parents really tries to help me |  |  |  |  |
| 4 | My parents are willing to help me make decisions |  |  |  |  |
| 5 | I can talk about my problems with my parents |  |  |  |  |
| 6 | My parents empathetically understand my concerns |  |  |  |  |
| 7 | My parents are always around when I am in need. |  |  |  |  |

**Self esteem**

| These are questions concerning your self-esteem. Just tick on the alternative answer in each question that you think better express you. | | | | | |
| --- | --- | --- | --- | --- | --- |
| S/N | Questions to assess the level of self esteem | Alternative responses  1= Strongly disagree  2= Disagree  3= Agree  4= Strongly agree | | | |
|  |  | 1 | 2 | 3 | 4 |
| 1 | On the whole, I am satisfied with myself |  |  |  |  |
| 2 | At times I think I am no good at all |  |  |  |  |
| 3 | I feel that I have a number of good qualities |  |  |  |  |
| 4 | I am able to do things as well as most other people. |  |  |  |  |
| 5 | I feel I do not have much to be proud of |  |  |  |  |
| 6 | I certainly feel useless at times |  |  |  |  |
| 7 | I feel that I am a person of worth, at least on an equal plane with others |  |  |  |  |
| 8 | I wish I could have more respect for me |  |  |  |  |
| 9 | All in all, I am inclined to feel that I am a failure |  |  |  |  |
| 10 | I take a positive attitude toward myself |  |  |  |  |

**Peer pressure**

| For each statement below, decide which sort of person you are. For each statement, mark your agreement as “X” the alternative that you think better explains you. | | | | | |
| --- | --- | --- | --- | --- | --- |
| S/N | Questions to assess the level of peer pressure | Alternative responses  1= Strongly disagree  2= Disagree  3= Agree  4= Strongly agree | | | |
|  |  | 1 | 2 | 3 | 4 |
| 1 | I often try what my friends do |  |  |  |  |
| 2 | I have a fear of saying no to what my friends encourage me to do |  |  |  |  |
| 3 | I frequently try to test all what my friends do just not because I believe in it but to stay on my friends good sides. |  |  |  |  |
| 4 | I often go with my friends and do what they do to keep them happy of me |  |  |  |  |
| 5 | If my friends and dorm mates invited me to do an activity, I always kindly do that |  |  |  |  |
| 6 | I feel ashamed when my friends feel unhappy of me in the case when I go different from their way |  |  |  |  |
| 7 | I feel what my friends do is always good to follow |  |  |  |  |

**Purpose of the internet use**

| The statements below describe the purposes/reasons why most people engaged in the internet. Mark “X” the alternative responses that you think better explains your reasons why you engaged in the internet. | | | |
| --- | --- | --- | --- |
| S/N | Which activity you most of the time perform using internet | Alternative responses | |
|  |  | 1= yes | 2 = no |
| 1 | To establish online friendships and chat with online friends/ social networking |  |  |
| 2 | To play different online games/ online gaming |  |  |
| 3 | To view and down load information concerning about football, listen music/entertainment |  |  |
| 4 | To download and read online books/articles for assignments and research related to my discipline /academics use |  |  |

**Thank you in advance for your time.**
