# Supplementary material for: Online activities as risk factors for Problematic internet use among students in Bahir Dar University, North West Ethiopia: A hierarchical regression model
Source: PLoS One. 2020 Sep 11;15(9):e0238804. doi: 10.1371/journal.pone.0238804 (PMC7485847; doi:10.1371/journal.pone.0238804)
Supplement: Attachment — Submitted filename: Response to reviewers.docx [file pone.0238804.s003.docx]

**Authors’ response**

**To the Editor**

Thank you for giving us this chance to revise and submit the manuscript. We have gone through all the concerns/comments of the editor and the reviewers. Many of the changes /improvements are highlighted yellow.

**Editor’ concerns**

1. Please ensure that your manuscript meets PLOS ONE's style requirements, including those for file naming.

*Response*: The revised manuscript is edited as to the requirements of PLOS ONE.

2. Occurrence of minor overlapping text with the following previous publications: it is corrected as follows..

*Response*: the observed overlaps are edited. The entire document was edited accordingly.

3: additional information about the survey to replicate the analysis, e. g., include a copy of the questionnaire.

*Response*: A copy of the questionnaire is attached (Additional file 1).

Concern 4: provide institutional address

*Response*: kerebiha@bdu.edu.et

Concern 5: Please upload the minimal anonymized data set necessary to replicate your study findings as either Supporting Information files.

Answer: The survey data are uploaded as supporting information

Concern 6: ORCID iD for the corresponding author is needed

*Response*: https://orcid.org/0000.0001-9225-2923

Reviewer 1

Thank you very much for the comments. We have gone through the comments and improved the document accordingly.

1. Justification for using hierarchical regression is not enough in the introduction.

*Response*: We conceptualized the phenomenon of PIU within the precept of Problem Behavior Theory. This theory specifies different levels of variables that may have differential effects on the behavior considered: some variables may have distal effects and others may have proximal effects. Previous researchers have reported a wide range of variables as risk factors of PIU. Though limited, recent studies also reported a number of activities that can be engaged in excessively online which may lead to symptoms similar to substance- related addictions. Thus, we hypothesized that the specific online activities are proximal and my significantly predict PIU when the socio-demographic (distal) variables are controlled.

2. The method of calculation for the required sample size seems to be one used for prevalence studies (assuming 50%). I do not think this study is one of those.

*Response*: Previous research on prevalence of PIU in Ethiopia is not available. We estimated the prevalence of PIU and assessed factors that contributed for the phenomenon. Thus, we assumed the prevalence as 50% to increase the sample size and improve the generalizability of the findings.

4. Was the Ethiopian IAT validated? If not, that is a limitation and needs to be mentioned.

*Response*: The IAT we used is not validated. We pilot tested it and the internal reliability (Cronbach's ) was .93. WE also stated it in the limitation section.

5 & 6. How were the online activities reported?

*Response*: In this study, students’ engagement in online activities was measured categorically as yes/no. Such binary scoring did not indicate the amount of internet use (time spent) hence likely to lead to inflated prevalence of each activity. This was described in the limitation section of the document.

7. The interpretation of the result that says something is more important than the existing variables because it improves the fit of the model should be revisited.

*Response*: The order of entry of the variables in the model was made based on the conceptualization of the phenomenon. And the interpretation was shaped as the online activity variables significantly predicted PIU when the socio-background variables were controlled.

8. The authors need to understand that they didn’t ‘examined the relative importance’ of risk factors of IA. Interpretation needs to be revised thoroughly.

*Response*: Model II included all the variables and indicates the values of adjusted odd ratios. A variable with the highest odds ratio is relatively highly correlated with the dependent variable. Accordingly, this was explained in the revised document.

**Reviewer 2**

Thank you very much for the comments. We have gone through the comments and improved the document accordingly.

1. Although it is an interesting study but a major issue is found for the statistical analysis. The authors must provide more details whether the required conditions were met for multiple regressions.

*Responses*: Assumptions for using logistic regression were checked. The existence of multicollnearity between independent variables was checked using tolerance and variance inflation factor (Additional file 2). Interaction effects among independent variables were also computed and the results indicated no interaction effects. These are included in the method section of the revised manuscript.

2. Concerns on Tables 1, 3 and other comments forwarded to improve readability and maintain consistency in reporting numbers,

*Response*: The revised manuscript is improved as to the comments.

3. When examining the risk factors by logistic regression, please add further explanation.

*Response*: It was explained in the methods part in the analyses section.

4. Is sex included for the model I analysis?
*Response*: sex was not included in the multivariate regression models. It was not found significant associated with the dependent variable at the bivariate analysis. Those variables with significant association in the bivariate analyses were included in the multivariate analysis.

5.year stayed in the University and age may overlap. Please provide more data for collinearity check.
*Response*: The existence of multicollineariy among independent variables was computed and the findings indicated no problem of multicollinearity (Additional file 2). This was presented in the methods par (data analyses portion).

6. Concern on clarity of the Nagelkerke ‘s R^2^ for models :

*Response*: This study investigated the roles online internet applications play on problematic internet use behaviors of undergraduate students. Hierarchical multivariate logistic regression models were used to assess the relationship between independent variables and outcome variable. The overall fit of each logistic regression model was assessed by using its Chi-square (model Chi-square and step Chi-square) as well as goodness-of-fit indices (_2 log likelihood) and improvement observed in the model with the predictors relative to the model that proceeded was assessed. In addition to indices of the overall model fit, Nagelkerke’s R^2^ was evaluated as an approximate estimate of the amount of variance in the dependent variable accounted for by the model. A model with the highest Nagelkerke’s R^2^ value is relatively better model in explaining the dependent variable. Such discussions are presented in the data analyses portion and in the result presentation sections, specifically predictors of PIU portion.

**Reviewer 3**

*General response for the reviewer*: Thank you very much for your critical review and thoughtful recommendations. We learned a lot from your suggestions. We attempted all your concerns as stated below and improvements are highlighted yellow in the manuscript.

**Comments in the introduction section**

1. Relevant and recent literature should be included in the introduction.

*Response*: The introduction part of the manuscript is improved as to the comments. Improvements are marked with yellow in the manuscript.

**Comments on the aims of the study**

1. Review the aim of the study and restate the hypothesis

*Response*: The aim of the study is edited and the hypothesis is restated as to the comments.

2. Regroup the background (control) variables and the predictor variables

Response: As to the comments, the variables in the study are regrouped into two- socio-demographic background variables (controls) and the online activities (predictor variables).

**Comments on methods of the study**

1. While deciding the sample size, the prevalence of PIU was assumed to be 50% in Ethiopia while the prevalence rate is very low worldwide

*Response*: Previous research on prevalence of PIU in Ethiopia is not available. We estimated the prevalence of PIU and assessed factors that contributed for the phenomenon. Thus, we assumed the prevalence as 50% to increase the sample size and improve the generalizability of the findings.

2. Concern regarding the measures of parental support and peer pressure

A. Parental supervision is completely different phenomenon than parental support

*Response*: In this study, we measured students’ perception of their parents’ support after they enrolled into the university- the relational aspect.

B. You were using the term “peer pressure,” but measured “resistance to peer pressure” –

Response: In this study, we measured peer pressure- the tendency of individual student to affiliate with like-minded friends. The tool is modified to fit this purpose.

3. Cut-offs used for Young’s Internet addiction test scores?

Response: The cut-offs used was same as suggested by Young.

4. Mention the response rate, the amount of missing values and how they were addressed.

*Response:* The total sample size for the study was 844 students. Data obtained from 32 students were not complete (had missing values), hence excluded. Data obtained from 812 students were used in the study. Thus the response rate was 96%. This information is included in the method part (described in the sample size and sampling procedure descriptions as well as in the limitation portion).

**Comments on Results**

1. There are significant associations between PIU and both the age and years spent at university. While these two variables are most probably also associated, one of the two associations with PIU could be artificial. Could you add another analysis that would shed light upon this?

*Response:* The existence of multicollinearity between independent variables was assessed. The data indicated no problems of multicollinearity among the independent variables. This was explained in the method part.

2. When comparing predictive models, you should report not only R^2^ and its changes, but also F-value (with significance), that shows how much (and if significantly) models differed

*Response*: Predictive models were compared employing the multiple binary logistic regression and the reported R^2^ s are values of Nagelkerke R^2^ (pseudo R^2^) and the significance of model improvement explaining PIU was explained by statistically significant in model Chi- Square (χ2 (11) = 329.605, P <0.001).

3. You could elaborate it in introduction and perform the analysis with self-esteem as a mediating variable between grades and IA.
*Response*: We understood that this was beyond the aim of the study and the statistical technique (binary logistic regression) employed to see the importance of online activities in explaining PIU while controlling other background characteristics.

**Comments on discussion**

1. The sources are not very recent and sometimes I missed the logic behind their inclusion.

Responses: Improvements are incorporated in the revised manuscript.

2. When discussing the prevalence, you should take into account the measurement of PIU.

Response: The discussion was presented considering the comments.

3. Discuss your results on the type of online activity with relevant studies

*Response*: It was incorporated in the revised document

4. Please be careful about the causality statements, especially in case of grades

*Response*: The concern was supportive in discussing the findings.

5. There are more recent sources dealing with IA and self-esteem

*Response*: Improvements are incorporated as to the comments.

6. Clarification/rewriting: how findings can be “novel” and at the same time “consistent with previous studies”?

*Response*: This was edited as to the comments.

7. When you discuss the effect of years spent at university (“seniority of students”), you should take into account the associations between PIU and age.

*Response:* The wald statistics in Table 3 indicated no significant association between the two variables when the roles of other variables were controlled.

8. The paragraph on the effect of parental support needs to be completely rewritten and accompanied by relevant sources.

*Response*: Improvements are incorporated in the revised document.

**Comments on limitations**

**1.** State response rate

Response: it is included in the revised manuscript.

**2.** You should clearly acknowledge the limitations

Response: The limitation part of the document is revised considering the comments and suggestions given by the reviewer.

**Comments given as minor issues**

*Response*: Comments given as minor issues by the reviewer were considered and improved accordingly.
