# Supplementary material for: Online activities as risk factors for Problematic internet use among students in Bahir Dar University, North West Ethiopia: A hierarchical regression model
Source: PLoS One. 2020 Sep 11;15(9):e0238804. doi: 10.1371/journal.pone.0238804 (PMC7485847; doi:10.1371/journal.pone.0238804)
Supplement: Attachment — Submitted filename: Response to Reviewers.docx [file pone.0238804.s004.docx]

**Responses to Reviewers**

**To the Editor**

Thank you for giving us this chance to revise and submit the manuscript. We have gone through all the comments of the reviewers. Many of the changes /improvements are highlighted yellow.

**Reviewer 1**

Thank you very much for the comments. We have gone through the comments and improved the document accordingly.

1. The interplay between independent variables and dependent variables needs to be explained.

***Response***: Variables entered in the analyses were selected based on the existing literature describing possible relationship with the dependent variable. Some authors reported that the individual and environmental factors (age, sex, academic achievement, parental support, peer pressure self-esteem) significantly related to PIU. Others differentiated between dependence to the internet and dependence on the internet and argued that majority of individuals with PIU use the internet as a medium of online activities, such as social networking, online entertainments, and gaming. And these variables are suggested as more important for PIU. These are explained in the manuscript (lines 104 – 112).

2. Concern related to sample size calculation: Why was a sample size calculation method for prevalence study used?

***Response***: The main purpose of the study was to learn the roles of online activities on the internet in predicting PIU. We need to know whether there was PIU and assess which variables (the control or predictor variables) better explain he variance of this phenomenon. Thus, we used the approach to estimate the sample size.

3. Either the effect size or the p-value should be taken as indicating relative importance between variables.

***Response:*** As it is suggested, reporting effect size or lower p-value indicates the relative importance of the independent variables in explaining the dependent variable. In the current study, use of the internet for social networking, online gaming, peer pressure, and grade point average had lower p-vales. These variables are relatively important in explaining PIU among students. Nevertheless, the interest in this study was to assess whether socio-demographic variables (controls) or the online activities (predictors) better explain PIU among students. The interest was to compare the models in explaining the outcome variable. Our results revealed statistically significant improvement in predicting PIU of students with the online activity variables after controlling the socio-demographic background variables.

**Reviewer 2**

Thank you very much for the comments. We have gone through the comments and improved the document accordingly.

1. Concern related to consistency of using terminology: using PIU instead of internet addiction.

***Response***: The manuscript was edited as to the comment.

**Reviewer 3.**

Thank you very much for your critical review and thoughtful recommendations. We learned a lot from your suggestions.

**Methods section**

1. Sampling: Concern related to knowing eligible participants based on inclusion/exclusion criteria and the response rate

***Response*:** There were 2010 students in the selected 10 departments. The sample size was proportionally allocated to number of students in each department and selected students were approached through section mentors. Written consent of individual participants was obtained after being fully informed of the study purpose and procedures. The inclusion criteria (reside in the university dormitory, used internet at least for six months, and not diagnosed with anxiety or depression disorders for a year) were communicated while students were completing consents. Fortunately, no student reported discordant information to be excluded from the study. Thus, all were eligible to participate in the study. During data cleaning, we identified that 32 students did not fully complete questionnaire, hence excluded from the study. Data obtained from 812 respondents (96.2% response rate) were reported in this study.

2. Measurement

A. Clarity on the assessment of peer pressure

***Response:*** Originally, the tool was developed to measure resistance to peer pressure. In this study, the scoring is reversed: higher score indicated higher vulnerability to peer pressure.

B. Cut-offs used for Young’s Internet addiction test scores?

***Response***: The cut-offs for Internet addiction test scores were modified as to the reviewer’s comments.

**Discussion section**

A.Concern related to prevalence of PIU.

***Response***: In the revised manuscript, the prevalence of PIU was discussed in line previous research reports those used similar measurement tool (Internet Addiction Test) as suggested by the reviewer (lines 331 – 339).

B. Concern related to whether the effects of online activities on PIU was independent of gender (chi-square test of association for the frequency of gaming and social networking in male and female students)

***Response***: In the revised manuscript, bivariate analysis of our findings revealed that female students were more likely to show symptom of PIU than their male counterparts. When the effects of other variables are controlled in the multivariate analysis, such difference failed to reach significant. The main purpose of the study was to learn the roles of online activities on the internet in predicting PIU. Gender was one of the control variables entered first in the regression model and the improvement of the second model with online activity variables in explaining the variance in PIU was assessed. Below is the distribution of male and female students by online activities assessed in the study.

| Online activities | Sex | | **χ^2^** |
| --- | --- | --- | --- |
|  | Male  n(%) | Female  n(%) |  |
| Social networking use | 406(75.9) | 207(74.7) | .132 |
| No use | 129(24.1) | 70(25.3) |  |
| Entertainment use | 408(76.3) | 190(68.6) | 5.531* |
| No use | 127 (23.7) | 87(31.4) |  |
| Academic works use | 393(73.5) | 183(66.1) | 4.838* |
| No use | 142(26.5) | 94(33.9) |  |
| Online game use | 119(22.2) | 56(20.2) | .443 |
| No use | 416(77.8) | 221(79.8) |  |

**Limitation section**

1. Concern related to limitations of Young’s Internet addiction test should be mentioned.

**Response:** The revised manuscript is improved as to the comments given by the reviewer (lines 416 – 423).

**Minor issues**

Language and editorial works

Response: A professor in English language teaching English as a foreign language edited the manuscript.
