# Supplementary material for: Online activities as risk factors for Problematic internet use among students in Bahir Dar University, North West Ethiopia: A hierarchical regression model
Source: PLoS One. 2020 Sep 11;15(9):e0238804. doi: 10.1371/journal.pone.0238804 (PMC7485847; doi:10.1371/journal.pone.0238804)
Supplement: Attachment — Submitted filename: Response to Reviewers.docx [file pone.0238804.s005.docx]

**Responses to Reviewers**

**To the Editor**

Thank you for giving us this chance to revise and submit the manuscript. We have gone through the comments of the reviewers. The changes /improvements are highlighted yellow.

**Reviewer 1**

Thank you very much for the comments. We have gone through the comments and improved the document accordingly.

1.  You should consider eliminating the word "relative" in the first line in the "Conclusion

***Response***: we changed as to the comment.

**Reviewer 3.**

Thank you very much for your critical review and thoughtful recommendations. We learned a lot from your suggestions.

1. Technical issues should be corrected (e.g. past tense while describing results - rows 251, 252 and elsewhere, consistency in replacement of IA by PIU - row 450).

***Response*:** Improvements are made as to the comments.
